# Supplementary material for: Microstructure, Physical and Biological Properties, and BSA Binding Investigation of Electrospun Nanofibers Made of Poly(AA-co-ACMO) Copolymer and Polyurethane
Source: Molecules. 2023 May 8;28(9):3951. doi: 10.3390/molecules28093951 (PMC10180346; doi:10.3390/molecules28093951)
Supplement: Supplementary file 1 [file molecules-28-03951-s001.zip › molecules-2378593-supplementary.pdf]

Supporting Materials .1

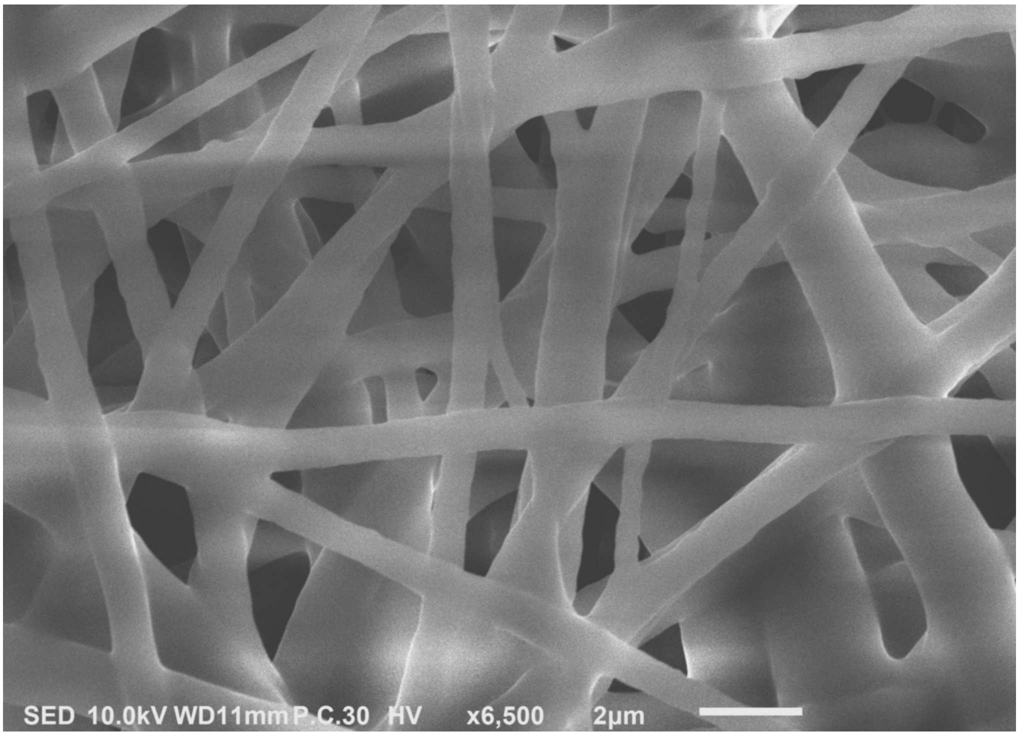

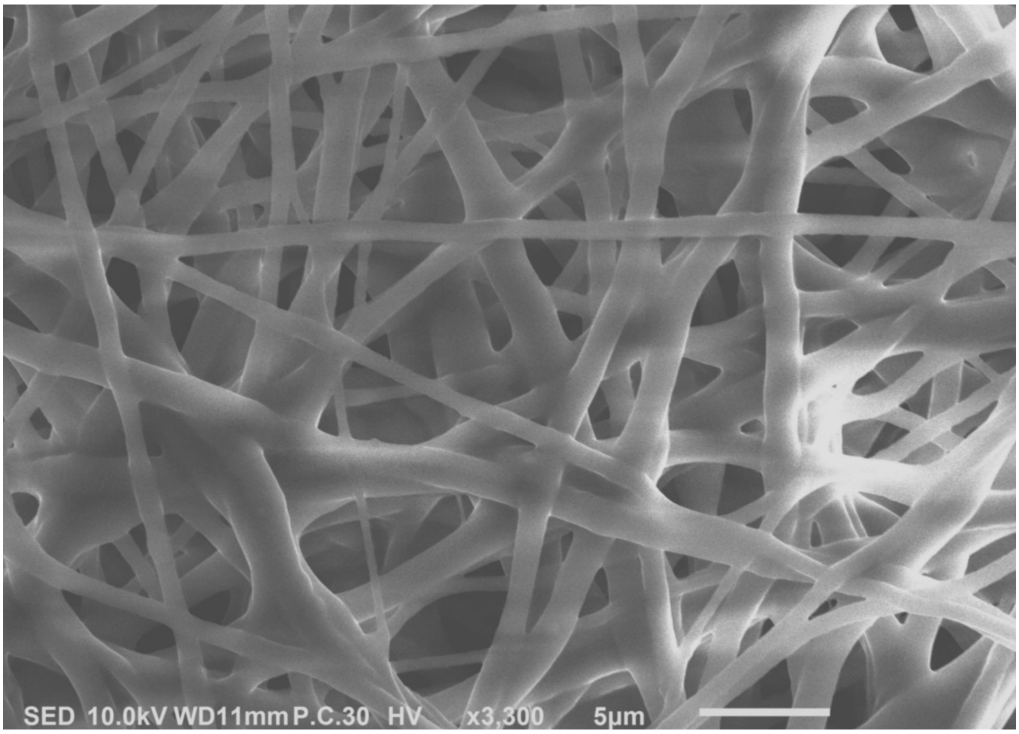

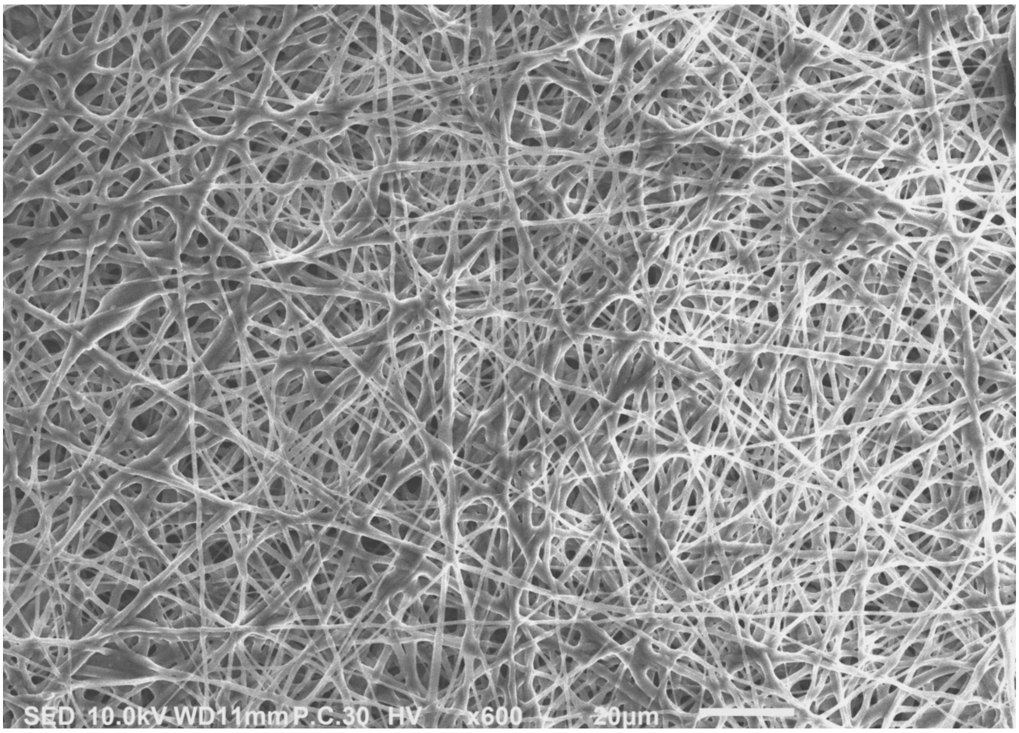
SED 10.0kV WD11mm P.C.30 HV x600 20µm

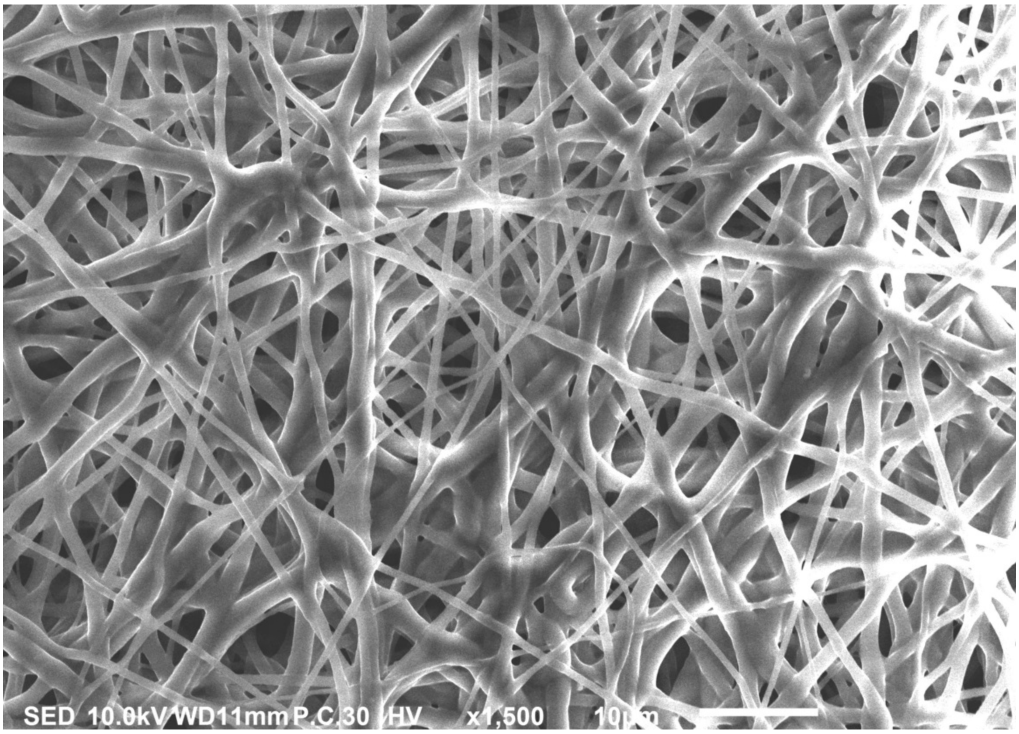
SED 10.0kV WD11mm P.C.30 HV x1,500 10µm

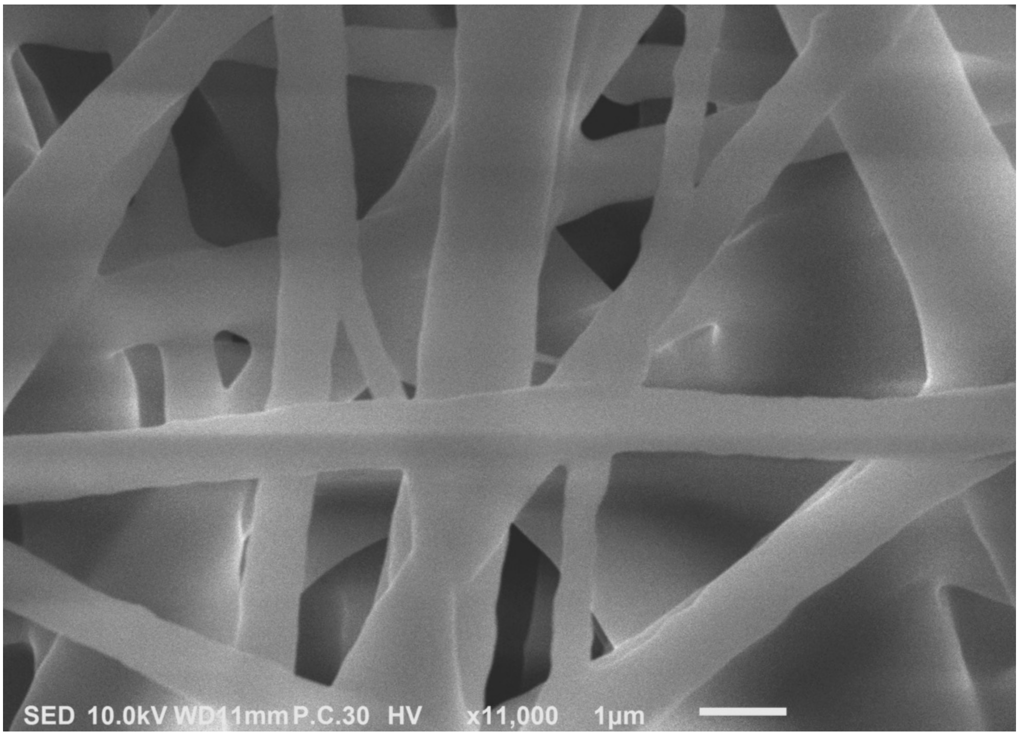

Figure S1. SEM images of the electrospun nanofibers **PU**.

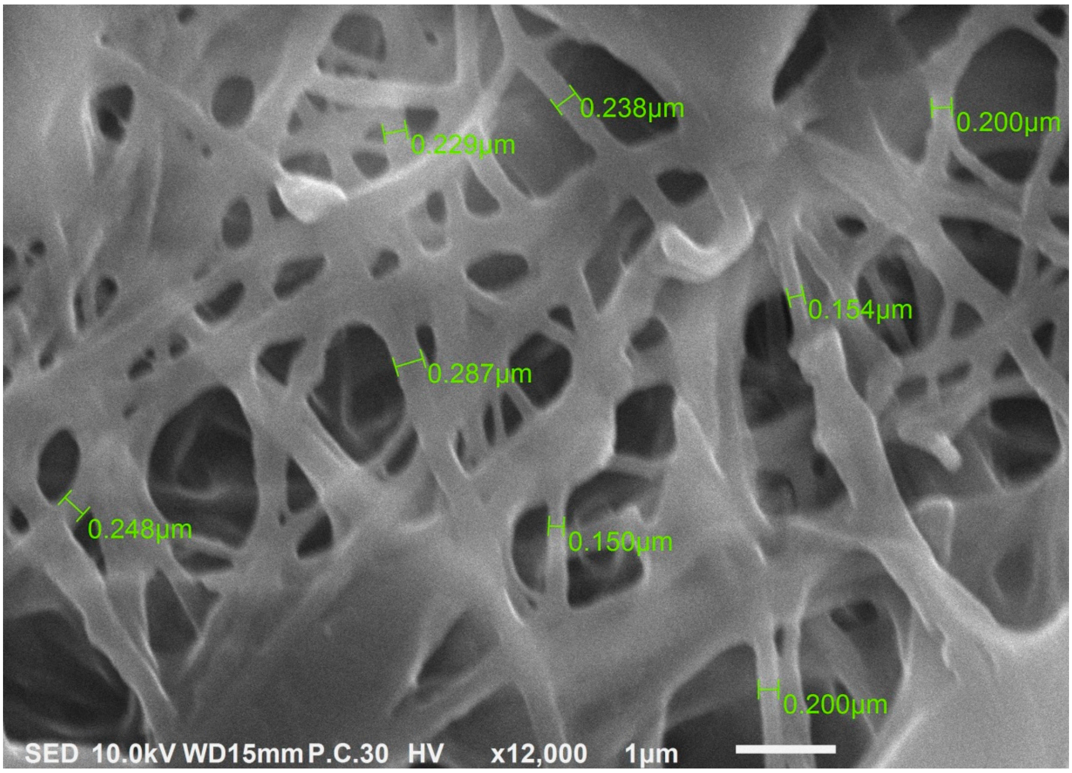

0.238μm
0.229μm
0.200μm
0.154μm
0.287μm
0.248μm
0.150μm
0.200μm
SED 10.0kV WD15mm P.C.30 HV x12,000 1μm

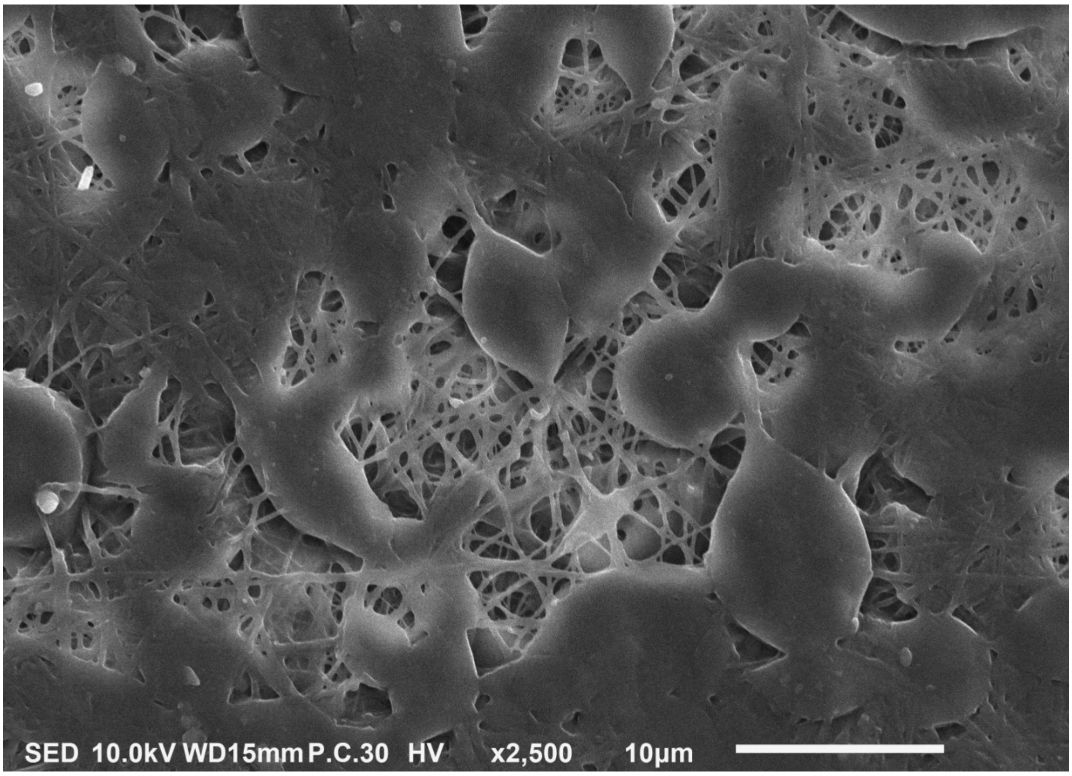

SED 10.0kV WD15mm P.C.30 HV x2,500 10μm

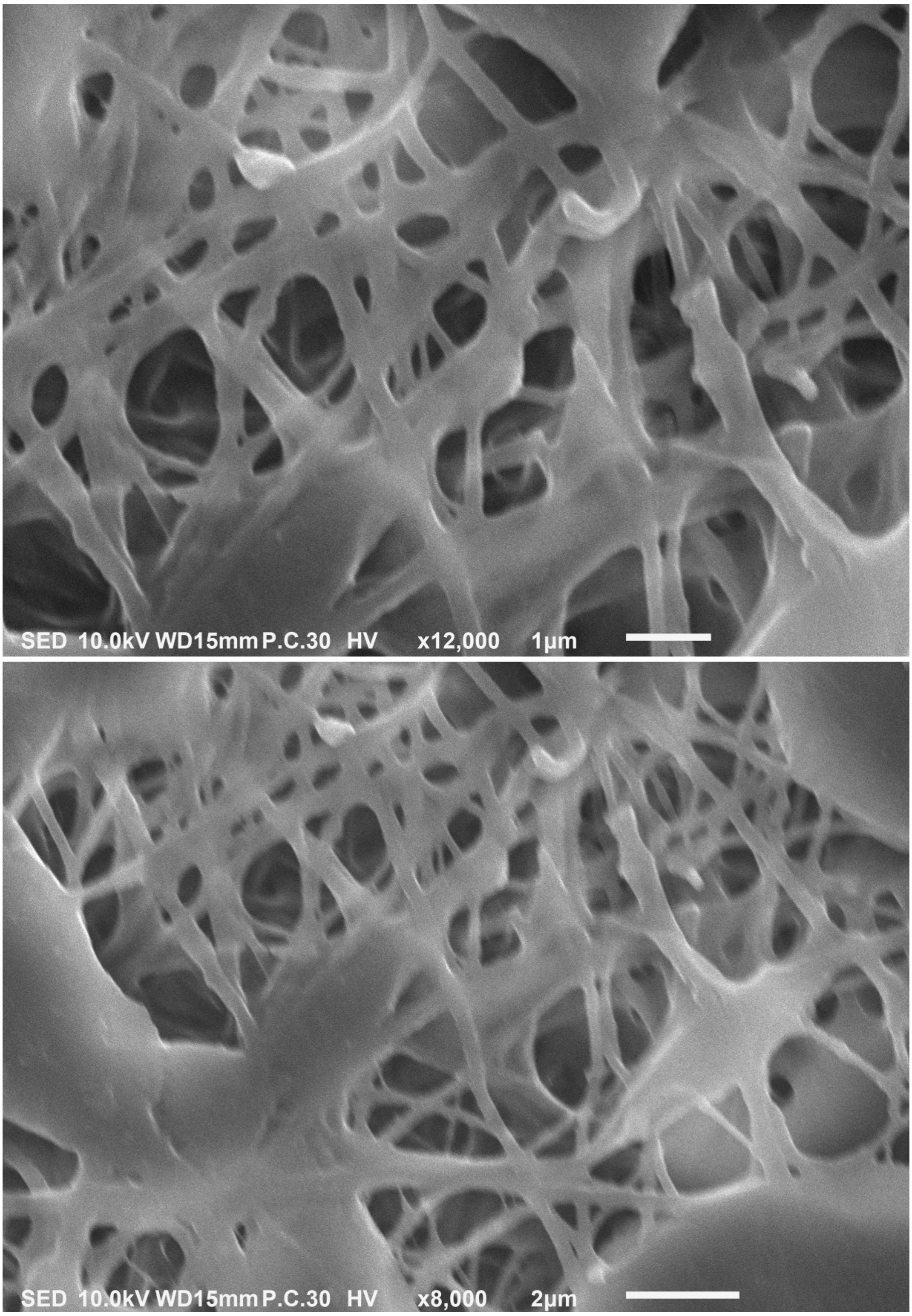

Figure S2. SEM images of the electrospun nanofibers **NF11**

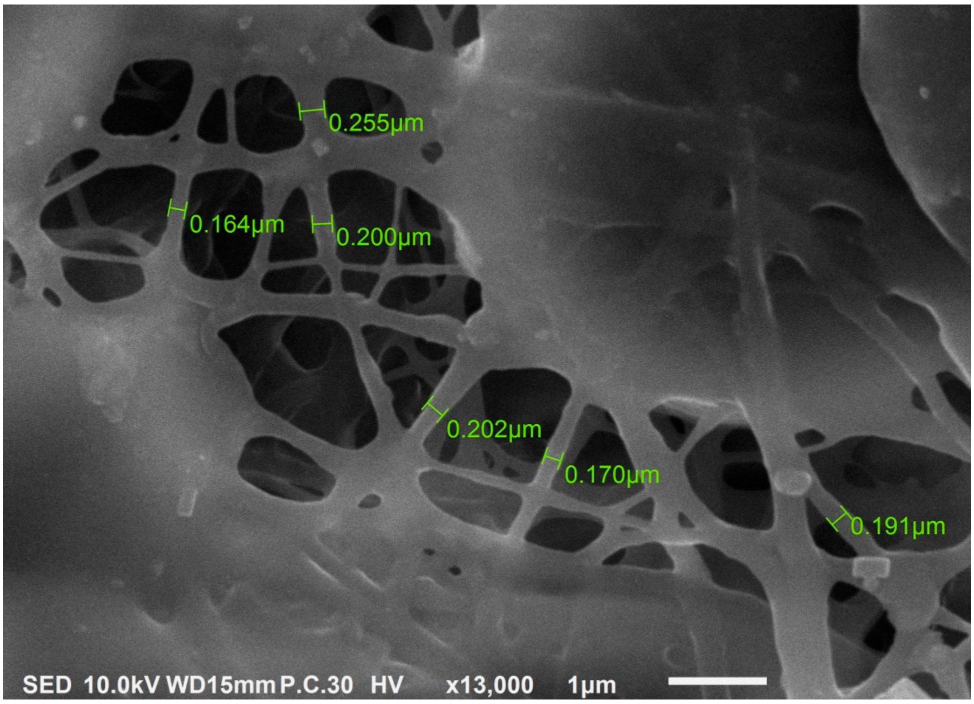
0.255μm
0.164μm
0.200μm
0.202μm
0.170μm
0.191μm
SED 10.0kV WD15mm P.C.30 HV x13,000 1μm

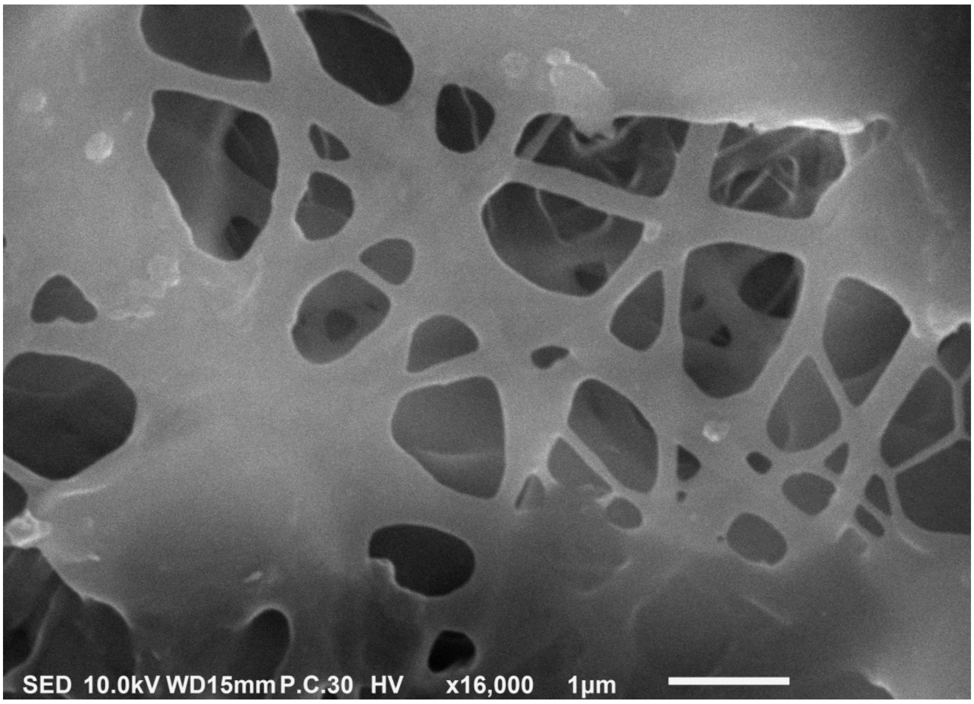
SED 10.0kV WD15mm P.C.30 HV x16,000 1μm

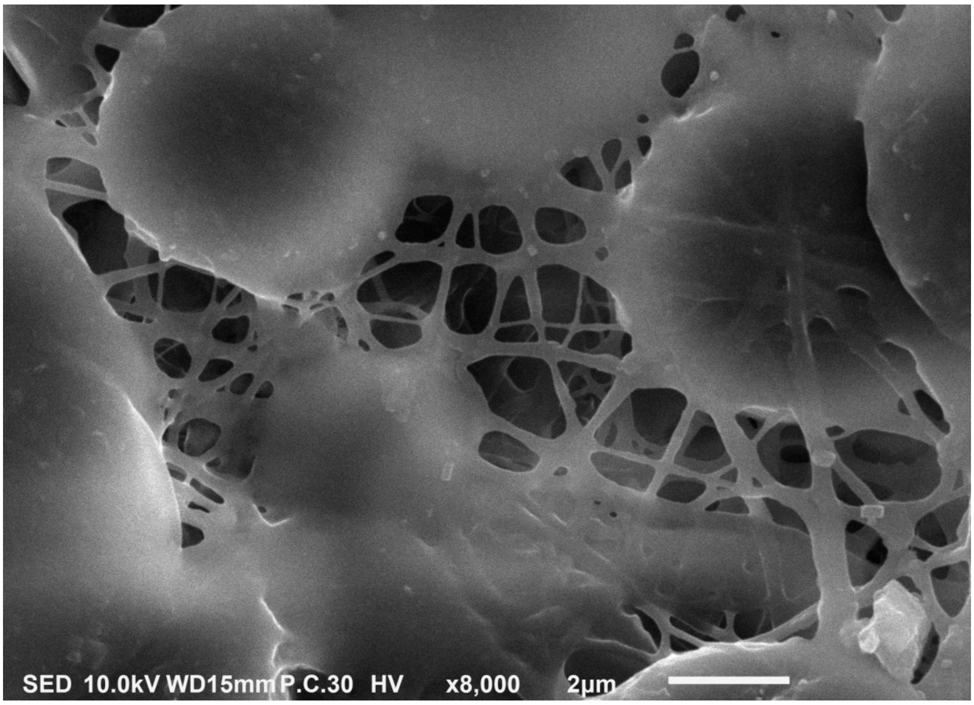
SED 10.0kV WD15mm P.C.30 HV x8,000 2µm

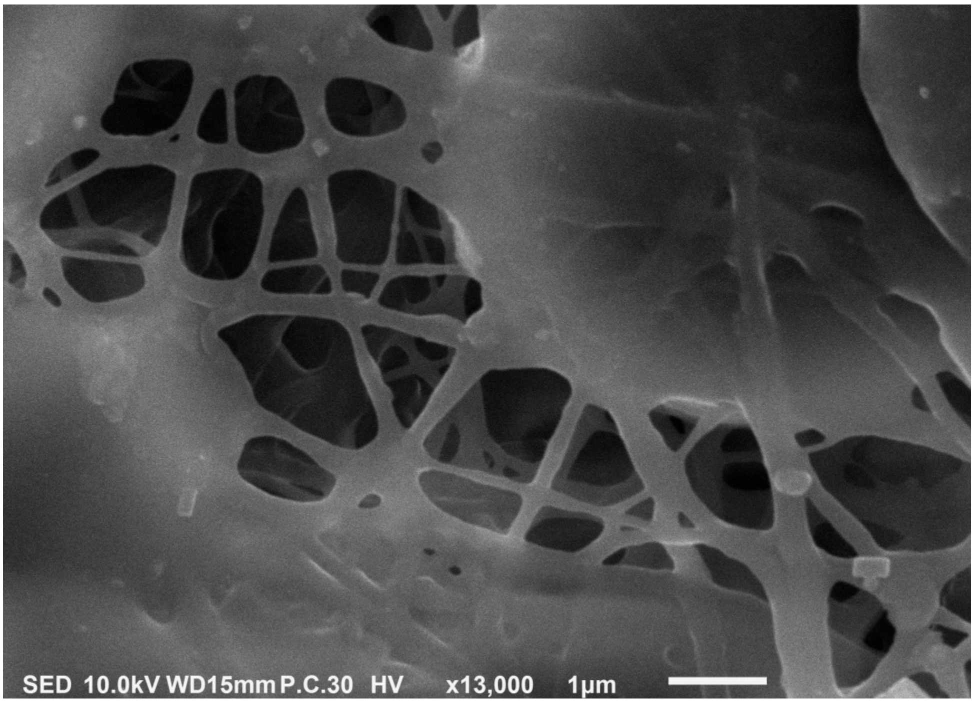
SED 10.0kV WD15mm P.C.30 HV x13,000 1µm

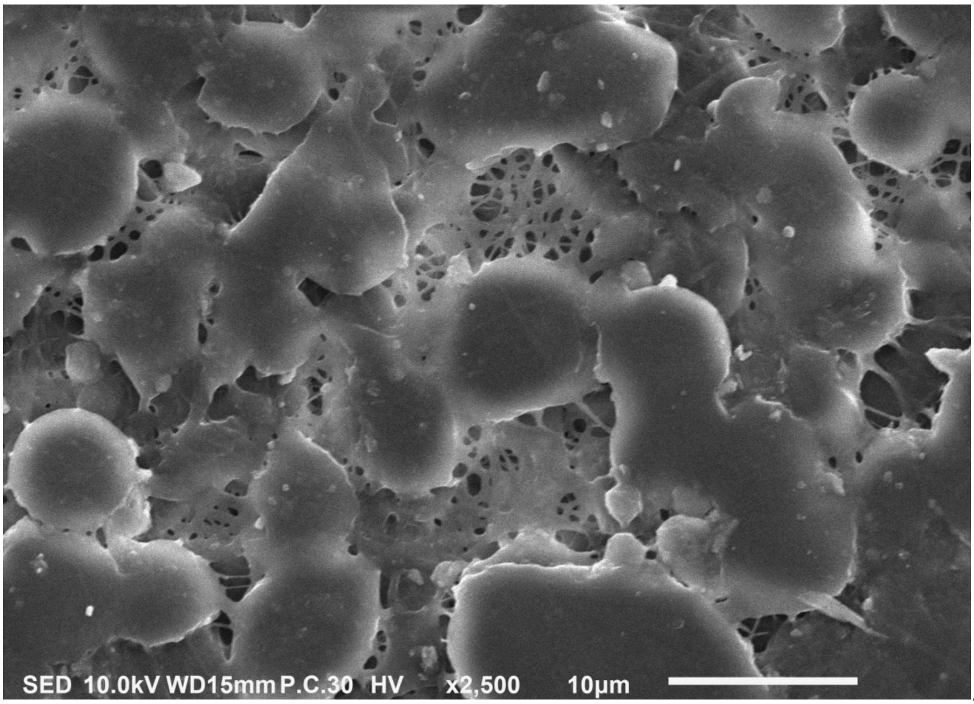

Figure S3. SEM images of the electrospun nanofibers **NF21**

Table S1. Tensile results of **PU**, **NF11** and **NF21** nanofibers.

Gauge Length (mm)	Width (mm)	Breadth (mm)	Area (mm^2)	Sample	Speed (mm/min)	Tensile Strength (MPa)	Young's Modulus (MPa)	Stain at Break (MPa)
20	10	0.025	0.25	NF11	2	14.97228613	39.02446197	9.153561181
20	10	0.03	0.3	NF11	2	14.28482108	38.09222654	9.285563161
20	10	0.035	0.35	NF11	2	18.25692774	60.83796882	9.102875727
20	10	0.165	1.65	PU	2	5.066446186	7.099307274	3.770019781
20	10	0.23	2.3	PU	2	4.905684152	9.664012301	2.634560679
20	10	0.25	2.5	PU	2	5.587143122	9.022896514	3.856163025
20	10	0.06	0.6	NF21	2	9.785045673	15.63562336	8.244864605
20	10	0.06	0.6	NF21	2	7.523199246	24.15903296	5.77640464
20	10	0.06	0.6	NF21	2	8.211512053	20.06515593	5.953748933
